# Mimickers of anterior uveitis, scleritis and misdiagnoses- tips and tricks for the cornea specialist

**DOI:** 10.1186/s12348-024-00396-z

**Published:** 2024-04-10

**Authors:** Sonny Caplash, Manuel Paez-Escamilla, Mark Westcott, Kunal K. Dansingani, Chad Indermill, Nacima Kisma, Eric Frau, Jose-Alain Sahel, Bahram Bodaghi, Vishal Jhanji, Marie-Helene Errera

**Affiliations:** 1 Department of Ophthalmology, University of Pittsburgh, 203 Lothrop Street, Pittsburgh, PA 15213 USA; 2 NIHR Biomedical Research Centre for Ophthalmology, Moorfields Eye Hospital NHS Foundation Trust, 162 City Road, London, EC1V 2PD UK; 3 Universite Libre de Bruxelles, Hopital Universitaire de Bruxelles, 808 route de Lennik 1170, Bruxelles, Belgium; 4 Department of Ophthalmology, Centre hospitalier National des Quinze-Vingts, Paris, France; 5 Department of Ophthalmology, Pitié-Salpêtrière University Hospital, Sorbonne Universités, F-75013 Paris, France

**Keywords:** Anterior uveitis, Scleritis, Episcleritis, Masqueraders, Drug induced uveitis, Immunotherapy, Antineoplastic, Ocular ischemic syndrome, Endophthalmitis

## Abstract

**Background:**

Anterior uveitis, inflammation of the anterior chamber and related structures, is a cohort of diseases that can present to almost any general or sub-specialty Ophthalmology practice. Its features classically involve anterior chamber cell and flare. Below the surface of these two signs exist a panoply of diagnoses.

**Body:**

The purpose of this review is to provide a general framework for diagnoses of anterior uveitis that are often missed as well as non-uveitic pathologies that often mimic anterior uveitis. Diagnostic deviation in either direction can have vision-threatening and rarely life-threatening consequences for patients. Using a comprehensive literature review we have collected a broad spectrum of etiologies of anterior uveitis that are easily missed and non-uveitic pathologies that can masquerade as anterior uveitis.

**Conclusions:**

We present a focused review on specific misdiagnosed anterior uveitis pathologies and some of the conditions that can masquerade as anterior uveitis and scleritis.

## Background

Anterior uveitis (AU) and anterior scleritis/ episcleritis, inflammations of the anterior chamber, sclera and related structures, are a cohort of diseases that can present to almost any general or sub-specialty ophthalmology practice. Their features classically involve anterior chamber cell, flare for anterior uveitis; and an inflammatory disorder of the sclera that may also involve the cornea, adjacent episclera, and underlying uveal tract for scleritis. Below the surface of these signs exist a panoply of diagnoses. Broadly speaking, the etiology for uveitis can be divided into infectious, inflammatory and autoimmune [1, 2].

The purpose of this review is to provide a general framework for diagnoses of AU/ scleritis that are often missed as well as non-uveitic pathologies that often mimic AU/ scleritis. Thus, the field of ocular inflammation possesses a significant challenge as many diseases may present as masqueraders and represent obscure diagnoses. Actually, when using the term “Uveitis Masqueraders”, most of these are simply different conditions with similar phenotypes or clinical appearance that mimic a chronic ocular inflammation. We must exclude neoplastic and non neoplastic ocular and systemic diseases uveitis that mimic chronic ocular inflammation. Among them, oncologic conditions such as lymphoma, hematologic malignancy, and paraneoplastic syndrome can present with ophthalmic manifestations. Infectious conditions, like Lyme disease, tuberculosis, syphilis, bartonella, Herpes and zoster virus have diverse ocular presentations, and treatment modalities. Patients who are immune compromised, either as a result of HIV infecting, cancer, or the treatment thereof, are at increased risk of infectious uveitis with atypical presentations. The growing field of drug-related uveitis and are also important considerations. The correct diagnosis often requires a comprehensive rheumatologic and ophthalmological evaluation, including: clinical history, multimodal imaging and systemic workup. Diagnostic deviation in either direction can have vision-threatening and rarely life-threatening consequences for patients. We reviewed the most common conditions that present as mimickers of AU, scleritis and conversely, inflammatory conditions that present as mimickers of AU, scleritis, and uveitis conditions with rare and diagnostically challenging etiologies.

Their typical and atypical presentations are reviewed as outlined below. Diagnostic strategies among the entities described below are also presented and discussed. (1.) Common vascular and neoplastic conditions that may present as anterior uveitis masqueraders; (2.) Misdiagnoses among uveitis/anterior segment inflammation entities (3.) Drug Related Uveitis. (4.) Masquerade presentations of anterior segment inflammation: e.g. Peripheral Ulcerative Keratitis (PUK) misdiagnosed for an infectious bacterial ulcer, (5.) Conditions that may present as scleritis masqueraders. (6.) Misdiagnoses among patients with scleritis.

## Method of literature search

The study was conducted according to the Preferred Reporting Items of Systematic Reviews (PRISMA) guidelines [3].

A literature search and subsequent screening of articles was conducted in 2022 by four authors (MPE, SK, GK, MHE). PubMed served as the primary database for the electronic literature search, although EBSCO and Cochrane were also surveyed. We systematically reviewed the available literature on neoplastic and nonneoplastic inflammatory masquerade syndromes. Literature searches were performed using electronic medical databases of the following keywords: Anterior uveitis OR anterior scleritis OR Neoplastic Uveitis OR Masquerader syndrome OR Uveitis mimickers OR medication induced uveitis OR Post-Vaccination Uveitis. The search timeframe was not limited by a specific date, but rather by the results of the articles retrieved.

The retrieved articles were initially screened by title and abstract, and articles with the relevant titles were then screened by full text using predefined inclusion and exclusion criteria. Inclusion criteria included 1) the paper must be written in or available in English and 2) the paper discussed the presentation and management of masquerade syndromes, inflammatory and infectious ocular diseases and uveitis. Exclusion criteria included 1) the paper concerned patients only with other inflammatory ocular diseases (episcleritis, scleritis) and AU, intermediate uveitis. 2) the paper did not clearly diagnose the patient with a masquerade syndrome. 3) citations were from grey literature. The full article was screened in cases where the relevance was unclear from the abstract. Relevant articles were ultimately compiled into a database and removed of duplicates. A total of 214 papers were finally selected.

No research ethics approval was needed for this study, as there were no human or animal participants included. The study protocol complied with the tenets of the Declaration of Helsinki.

## Common vascular and neoplastic conditions that may present as anterior uveitis masqueraders

Anterior uveitis (AU) has a variety of infectious, inflammatory and autoimmune etiologies. However, data show that up to 38-88% of all acute anterior uveitis cases are diagnosed as idiopathic, depending on geographic location [4, 5]. While there may be certainly a variety of uveitic presentations that are idiopathic in nature, there are several AU etiologies that are frequently misdiagnosed as idiopathic and are highlighted here.

## Neoplastic conditions masquerading as anterior uveitis

Although rare, leukemic infiltration of the anterior segment can mimic anterior uveitis. One study found only this type of leukemic infiltration in 0.5%–2.5% cases of leukemic relapses [6].

Beyond leukemia, other malignancies have been reported: metastasis in the case of lung, gastrointestinal and breast carcinoma, primary intraocular lymphoma (PIOL) [7, 8], systemic non-Hodgkin lymphoma metastatic to uveal structures within the eye, primary uveal melanoma (ciliary body), and retinoblastoma [9–16].

The most common type of lymphoma is Non-Hodgkin lymphoma (NHL). If it involves the eye, it usually localizes to the adnexal structures or the posterior segment, including the vitreous cavity, retina and uveal tissue [13].

Other lymphomas, like Burkitt lymphoma (BL), are associated with Epstein-Barr virus (EBV), with few reports of BL masquerading as anterior uveitis. Patients often present with orbital invasion, cranial neuropathy, optic neuropathy, and cavernous sinus infiltration with ophthalmoplegia. When it presents as anterior uveitis; eye redness, pain, photophobia and blurry vision are common complaints [17, 18]. Early in the disease course, dilated episcleral vessels, anterior chamber hyphema or hypopyon and circumferential engorgement of the iris can be seen [18, 19]. Examples in literature show: one eye with a non-granulomatous AU with vitritis and cystoid macular edema secondary to a mixed cellularity Hodgkin lymphoma, one eye with recurrent hyphema secondary to an iris mass from a mantle cell lymphoma, initially thought to be a UGH syndrome (ie., Uveitis-Glaucoma-Hyphema syndrome), and one case of peripheral non Hodgkin lymphoma presenting with pseudohypopion and iris nodules. Biopsies of the anterior segment lesions showed the intraocular tumor involvement [11–13].

In our experience, patients with hematologic malignancies can rarely present with anterior uveitis. In a case series we published of ocular involvement in six patients with hematological malignancies, we described one patient who had iris plasmacytoma and developed an anterior uveitis as a secondary presentation. The other patients in our series presented with a posterior ophthalmic involvement as pseudo-panuveitis or scleritis [20].

### Diagnostic implications

Involvement of the iris and anterior chamber is a recognized but unusual manifestation of leukemia. Although vitreal, uveal, and orbital involvement of NHL are widely recognized, anterior chamber involvement is less frequent. The differentiating features between hypopyon of inflammatory origin and tumoral pseudohypopyon have been very interestingly outlined by Evereklioglu et al. [21] In a pseudohypopion, the perilimbal area of the eye is not dusky red but rather white. Macroscopic signs to the naked eye shows that the anterior chamber meniscus is heaped up at its edges. These anterior chamber collections contain neoplastic cells, such as the authors also find in their unique case upon an aqueous tab, which revealed the presence of atypical lymphoblast cells [22]. Tumoral pseudohypopyon contains a lower concentration of fibrinous exudate which is mobile in the direction of leaning which is a pathognomonic feature of “tumoral pseudohypopyon”. Finally, a pseudohypopyon is “blood-streaked pinkish” and not white-colored [21].

Ocular biopsies (anterior chamber, vitreous or orbital), depending on the location of the malignant ocular lesion, and imaging of the eye by brain MRI enables the diagnosis. Of note, we would recommend discussing with the hematology services, who can perform biopsy of the bone marrow for final leukemia diagnosis since diagnosis involves integrating the clinical features, sites of involvement (predominantly peripheral blood, bone marrow, liver, and spleen), and cellular characteristics, including morphology and immunophenotyping. Biopsy of the conjunctival lesion when associated with cells in anterior and posterior segments can show a nonspecific, mixed inflammatory infiltrate and a granuloma but no malignant cells [23]. Chronic myeloid leukemia can be confirmed by hematological investigations, i.e., blood smear revealing increased leucocyte count with presence of abnormal cells (myelocytes, band forms, and promyelocytes) and subsequent bone marrow trephine biopsy [24] rather than by cytopathology of the hypopyon (predominance of lymphocytes and few plasma cells) [25]. Pseudo-hypopyon with isolated anterior segment involvement is a rare clinical presentation of intraocular B-cell lymphoma [26]. A high index of suspicion along with insisting on anterior chamber sampling for cytology and flow cytometry analysis are essential to establish the diagnosis [26]. As discussed by Ramani et al., cytological examination of rapidly transported, unfixed vitreous specimens is considered the gold standard in exclusion of intraocular lymphoma in patients with idiopathic steroid resistant chronic uveitis. The majority of vitreoretinal lymphomas are of a DLBCL histologic subtype, though occasionally T-cell lymphomas can occur. They usually express CD19, CD20, CD22, PAX5, BOB.1, and OCT2 [27].

### Vascular disease: Ocular Ischemic syndrome

Ocular Ischemic Syndrome (OIS) describes a constellation of clinical sequelae due to chronic, severe hypoperfusion in the setting of significant ipsilateral carotid or ophthalmic artery disease [28–33]. The classic etiology for this syndrome is vascular occlusion in the setting of severe atherosclerosis, typically greater than 90% occlusion of the ipsilateral carotid artery [29–31]. Other less common occlusive etiologies include Moyamoya [34, 35], giant cell arteritis [36–38], Takayasu arteritis [37, 39–44], optic disc melanoma [38] and radiation damage [44]. Patients may have orbital pain typified as a dull aching pain which can mislead the ophthalmologist for an inflammatory eye condition [30, 31, 45]. The posterior manifestations of mid-peripheral intraretinal hemorrhages and fluorescein angiography frequently displays delayed or patchy choroidal filling, retinal vascular staining and prolonged retinal arteriovenous transit time [34]. OIS can present as anterior uveitis in 18% of the patients [28, 30–32, 45–48]. In the anterior segment, rubeosis iridis and, neovascular glaucoma can be seen [32, 34]. Patients can present with anterior chamber cell and flare [34, 36, 46, 49]. Though it is important to note that cells and flare might co-occur in patients with OIS that also have rubeosis iridis [30]. The crux of its masquerading presentation lies in initial symptoms of ocular pain with anterior chamber cell and flare. However, given the cerebrovascular implications of severe carotid stenosis, a delay in diagnosis can have significant ramifications [28].

### Diagnostic implications

Atherosclerotic risk factors including hypertension, diabetes, prior cerebrovascular accident (CVA), coronary artery disease increase suspicion to a diagnosis of OIS when subacute to acute vision loss. Iridis rubeosis in the setting of posterior pole ischemia as represented by retinal artery attenuation with venous dilation and scattered dot hemorrhages can differentiate OIS from classic anterior uveitis.

### Infectious: syphilis

**Fig. 1:**
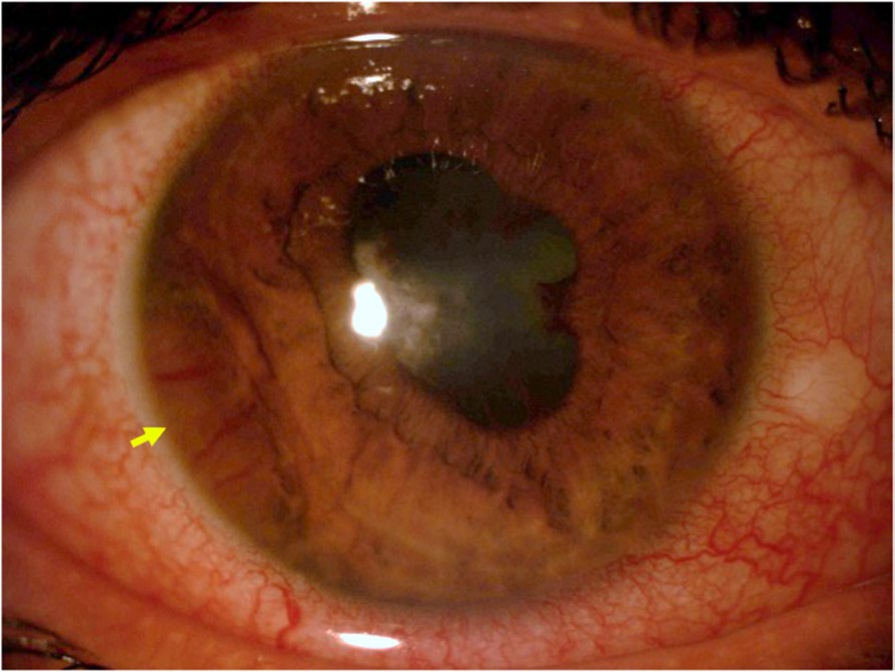
Slit-lamp photograph of a syphilitic gumma of the iris, a rare manifestation of tertiary syphilis

Ocular manifestations of ocular syphilis vary widely, but it most commonly presents as uveitis. It can present as any anatomic classification of uveitis with published reports documenting cases of anterior, intermediate, posterior and pan- uveitis. Despite the commonly held association between posterior uveitis and syphilis, it is important to note that syphilis is known as the great imitator and there are documented cases of anterior uveitis as well [50]. One case series found that 16.4% of ocular syphilis presented as anterior uveitis, 90% of which were in HIV positive patients [51]. We studied 21 cases (29 eyes) of ocular syphilis, finding only one patient that was affected with anterior uveitis representing 3.5% of the uveitis presentations [52] (Fig. 1). Most of the patients presented instead with a posterior uveitis in the form of posterior placoid chorioretinitis (58%) [53]. In our experience with a larger retrospective multicentric analysis of 95 patients treated for syphilic uveitis, 10 eyes, representing 14% of the cases, showed features of isolated anterior uveitis at presentation [54].

### Diagnostic implications

In the non-specific AU patient, consider syphilis testing.

### Post surgery: differential diagnosis of endophthalmitis after cataract surgery

#### Toxic Anterior Segment Syndrome (TASS)

Toxic Anterior Segment Syndrome (TASS), delayed-onset (chronic) endophthalmitis in the post-operative setting, retained lens material or lens-induced uveitis, de-hemoglobinized vitreous hemorrhage, and post-operative migration of triamcinolone in the anterior chamber are among the differential diagnosis for post-operative inflammation, presenting diagnostic and therapeutic challenges [55, 56].

Toxic anterior segment syndrome (TASS) is a syndrome that describes sterile anterior segment inflammation. It is most commonly associated with cataract surgery though there have been associations with other anterior and posterior segment surgeries [57–61]. The pathophysiology of TASS centers on the breakdown of the blood-aqueous barrier due to inflammation, specifically a breakdown in the tight junctions in the ciliary body and iris epithelium that separate the anterior chamber from the vasculature. Specific insults related to chemicals used in instrument processing cause an immune response resulting in anterior chamber inflammation [62]. Classically, it is known to present within 12-48 hours of recent intraocular surgery. Patients with TASS classically exhibit diffuse, “limbus-to-limbus” corneal edema. Anterior chamber inflammation with cell, flare, KPs and fibrin deposition are also found. In the acute setting, patients with TASS often exhibit a fixed and dilated pupil secondary to iris ischemia. This is also associated with decreased intraocular pressure due to decreased aqueous humor production.

Generally, TASS is a clinical diagnosis, made in the setting of a history of recent intraocular surgery, pertinent exam findings and laboratory data supporting a sterile inflammatory process.

#### Delayed-onset (chronic) endophthalmitis

While also typically infectious in etiology, it is distinct from acute infectious endophthalmitis in that it presents with recurrent episodes of low grade inflammation, 6 weeks or more after surgery [63]. Classically, a white plaque is seen between the intraocular lens and the posterior capsule. Over time, patients can develop hypopyon and vitritis. It can have both indolent bacterial or fungal causative organisms, with *Propionibacterium species* accounting for the majority of cases (41 to 63%) followed by coagulase-negative *Staphylococcus, Corynebacterium, cutibacterium acnes,* and others*. Aspergillus species, Candida species,* and *Curvularia lunata* are among the implicated fungal etiologies [63, 64]. Delayed-onset endophthalmitis cases are often managed by intraocular cultures (aqueous humor, and or vitreous) followed by repeated intravitreal antibiotics. Options for treatment of bacterial chronic bacterial endophthalmitis include diagnostic and therapeutic combined vitrectomy (PPV), intraocular antibiotics injection (IOAB), total capsulectomy, and removal or exchange of the intraocular lens (IOL). Current approach for chronic fungal postoperative endophthalmitis includes diagnostic and therapeutic PPV, intravitreal amphotericin or voriconazole, and a prolonged systemic antifungal drug [64].

#### Retained lens material or lens-induced uveitis

Lens associated uveitis represents another cause of post-operative inflammation. Disruption of the lens capsule results in a phacoantigenic uveitis, in which there is a granulomatous immune reaction centering on the site of lenticular injury and disruption. Leakage of lens protein often occurs in the setting of a hyper-mature cataract and results in a phacolytic uveitis as macrophages react to the dispersed protein. The resulting macrophage response results in a clogged trabecular meshwork and an acute increase in intraocular pressure.

In phacoantigenic uveitis, patients typically present with sudden or insidious ocular pain, redness, decreased visual acuity, and photophobia. In phacolytic uveitis patients present with acute onset of ocular pain, redness, and worsening vision [65–68].

Patients with phacoantigenic uveitis will present with an anterior uveitis with KPs, mild to moderate anterior chamber reaction and anterior vitreous inflammation without fundus involvement. Elevated IOP and posterior synechiae are also commonly present. Patients with phacolytic uveitis have conjunctival hyperemia, corneal edema, protein deposits (frequently mistaken for KPs) and anterior chamber cell and flare.

### Diagnostic implications

Differentiating causes of post-operative inflammation in patients can be difficult with specific ramifications to the direction of treatment. TASS can, in many ways, mimic infectious endophthalmitis, specifically chronic endophthalmitis, whose time course of weeks to months overlaps with TASS [2, 63, 64, 69]. Of the two diagnoses, it is reasonable to initially rule out chronic post-operative endophthalmitis, especially given the likely surgical management required. Careful follow-up for resolution can parse TASS from chronic endophthalmitis, which will not typically resolve with corticosteroids over time [62–64, 69–72]. Lens-associated uveitis related inflammation can be triggered in the setting of mature cataract lenticular leakage or disruption of lens capsule intraoperatively. In some instances, inflammation from severe AU may cause a sterile hypopyon that mimics the appearance of an infectious endophthalmitis. Management entails a thorough work-up to exclude an infectious etiology prior to initial treatment with topical and/or systemic steroids [73]. Because of the post-operative clinical context and the common presenting ocular findings, the use of microbiological samples is a key differentiator. In other words, chronic postoperative endophthalmitis should always be in the differential of recurrent postoperative inflammation and vitreous or aqueous samples should be sent for analysis early enough.

Below we describe some entities that are uveitis/ anterior segment inflammation/ scleritis related but are commonly misdiagnosed.

## Misdiagnoses among Uveitis/ Anterior segment inflammation entities

### Tubulointerstitial Nephritis and Uveitis (TINU) syndrome

Tubulointerstitial Nephritis (TIN) and Uveitis (TINU) is a multi-system pathology of unknown cause that commonly presents as an acute-onset bilateral, non-granulomatous AU with accompanied acute nephritis [74]. Eye inflammation can manifest in different anatomical forms. The most common exam findings include anterior chamber cell (65%) and flare, and conjunctival injection [75]. Occasionally keratic precipitates and vitreous cell can be seen as well. Complications of TINU include posterior synechiae, optic disc swelling and cystoid macular edema [76] and involvement of the vitreous or choroid may also be present [77–79]. We have recently published a case of AU and bilateral papillitis associated with TINU showing that it remains a common diagnosis among children and adolescents [80].

Key criteria for TINU have been updated by the SUN working group in 2021 and includes anterior chamber inflammation and evidence of tubulointerstitial nephritis with either (1) a positive renal biopsy or (2) evidence of TIN and an elevated urine β-2 microglobulin [81].

Since patients with TIN may be asymptomatic or exhibit nonspecific symptoms (fever, abdominal pain) that do not lead to kidney function tests being performed, a diagnosis of TINU may be significantly delayed or unrecognized even after the onset of uveitis symptoms and ophthalmological assessment [82–84]. Mandeville et al. noted that ocular symptoms were concurrent with systemic symptoms in only 15% of cases; in 21% of cases, uveitis occurred before systemic symptoms, occurring up to two months beforehand; in 65% of cases, uveitis occurred after systemic symptoms with a median time of onset being 3 months, though it was noted to extend up to 14 months [76].

It is often suggested that most incidence and prevalence figures of TINU are likely to be under-estimates. For example, Mackensen et al. identified that in a cohort of 1985 patients, 26 had been diagnosed with TINU during routine care (prevalence of 1.3%). However, on subsequent analysis, a further 7 patients who had been labeled idiopathic were found to have features consistent with TINU based on the criteria of typical bilateral sudden onset AU with renal dysfunction (total prevalence of 1.7%). They also identified that there were a further 18 ‘idiopathic’ pediatric cases in which the uveitis was typical for TINU but in whom there had not been adequate laboratory investigations to rule in or rule out the diagnosis, leading to the possibility that the real prevalence is even higher [76, 82].

### Diagnostic implications

The multi-system presentation of TINU can mimic other auto-immune pathologies including sarcoidosis, Sjögren’s syndrome, systemic lupus erythematosus, granulomatous polyangiitis, Behcet’s disease, tuberculosis and syphilis [76, 85]. The inconsistency of presentation timeline among cases can compound the diagnostic difficulty, more than half of the patients develop ocular symptoms after the acute interstitial nephritis [76]. This diagnosis should be promptly suspected in young patients presenting with either uveitis or TIN. A definite diagnosis is confirmed through renal biopsy.

### Immune reconstitution inflammatory syndrome

Immune Reconstitution inflammatory syndrome (IRIS) is observed in patients who have a diagnosis of AIDS with opportunistic infections in the setting of Acquired Immunodeficiency Syndrome (AIDS) with recovering CD4+ T cell counts on Highly Active Antiretroviral Therapy (HAART). While IRIS generally a broad spectrum of presentation, its ocular manifestation is most classically seen in patients with CMV retinitis and presents as uveitis. Ocular presentations of IRIS are termed immune recovery uveitis. The most common presenting signs are cystoid macular edema, epiretinal membrane, papillitis, and vitritis. However, there have notably been published cases of anterior segment inflammation. A review of Jabs et al. on ocular complications in patients with AIDS found that a significant proportion of those with immune recovery uveitis (IRU) had some degree of anterior inflammation, with up to 6.6% having posterior synechiae [86]. There were also a small proportion of patients with keratic precipitates on exam.

### Diagnostic implications

Patients with AIDS are at a high risk for a diverse range of opportunistic infections. Consideration of IRU in HIV+ positive patients with uveitis is critical in managing CMV-related therapy and appropriate initiation of topical corticosteroids.

### Cytomegalovirus (CMV)- related anterior uveitis

**Fig. 2:**
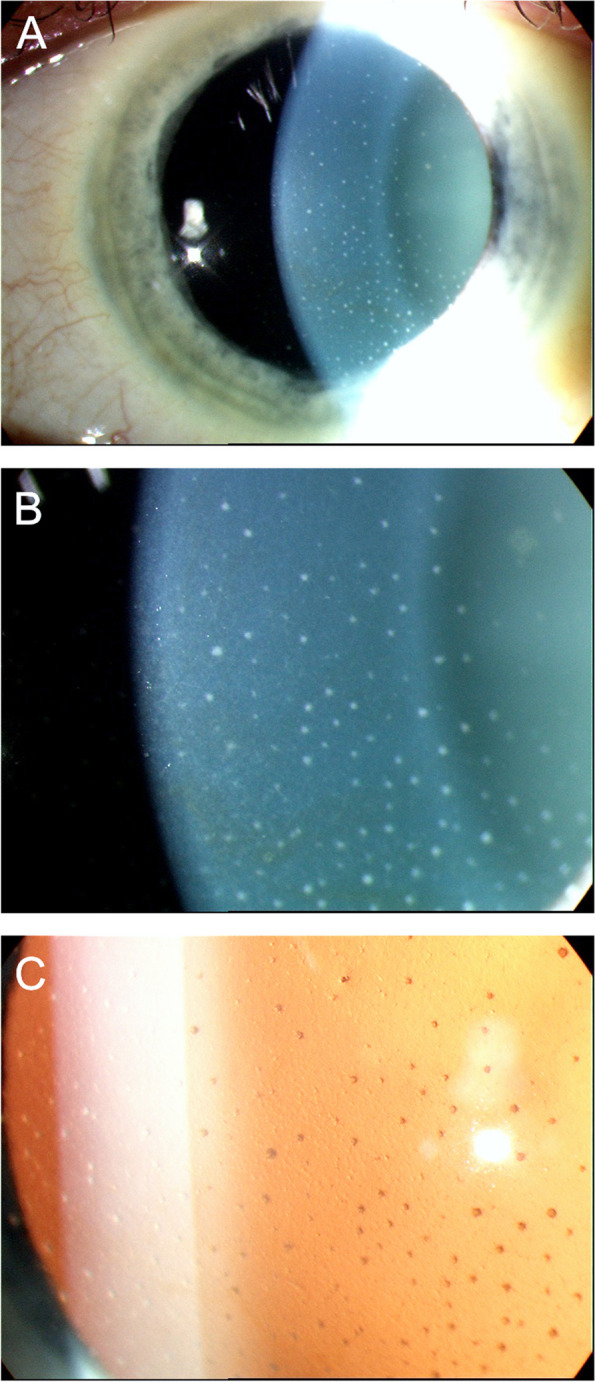
Slit-lamp photograph of an eye with Fuchs' heterochromic iridocyclitis. **A** Low magnification showing the typical stellate keratic precipitates, which are better delineated in high magnification in **B**) and retro-illumination in **C**)

**Fig. 3:**
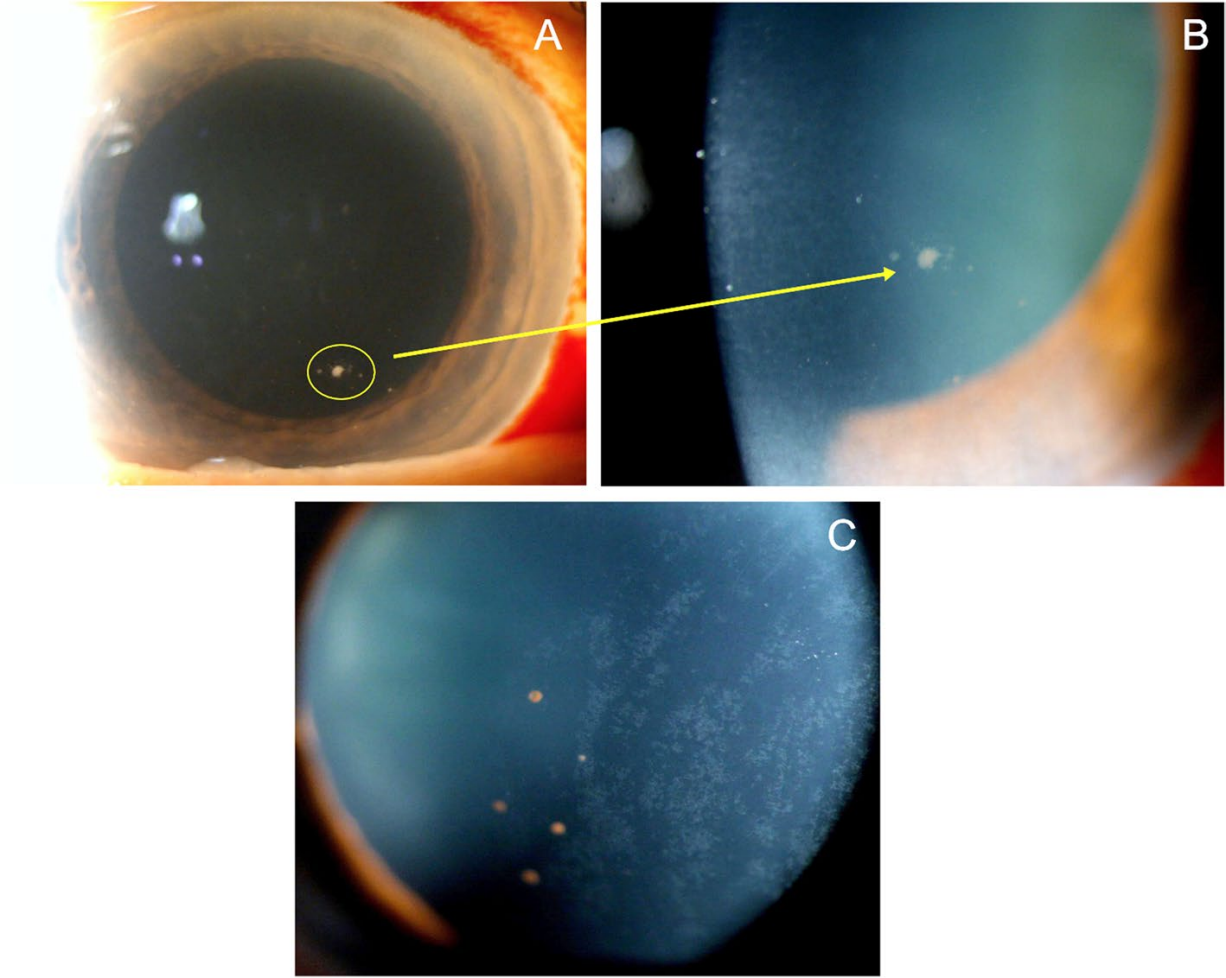
**A** Slit-lamp photograph of a coin-shaped keratic precipitate in the left eye of a 72-year-old female with Cytomegalovirus anterior uveitis. Diagnosis was confirmed by real-time PCR (RT-PCR) on aqueous humor samples. **B** Magnified image of the coin- shaped keratic precipitate. **C** Note in her right eye, few coin-shaped keratic precipitates

In immunocompetent patients, CMV presents as recurrent, acute or chronic, occasionally bilateral, AU often with associated ocular hypertension, and corneal endotheliitis [2, 87, 88], however it can have a broad spectrum of presentation. It has been documented to present as Posner-Schlossman Syndrome (PSS), an acute, recurrent hypertensive AU with few granulomatous keratic precipitates [88], and Fuchs uveitis syndrome (FHI), a chronic hypertensive AU with moderate anterior chamber reaction and diffuse, fine stellate keratic precipitates often involving the entire endothelial surface (Fig. 2). This later presentation appears to be more common in older patients, with a mean age of 65 years old [89]. Other forms include a chronic hypertensive AU with few, inferiorly-located, brown keratic precipitates and corneal endotheliitis with coin-shaped lesions (Fig. 3) or linear keratic precipitates (KPs) [87, 90].

Of note, Fuchs heterochromic iridocyclitis was once thought to be a non-infectious, idiopathic entity. Infection with herpes simplex virus, ocular toxoplasmosis, cytomegalovirus, rubella virus, and other viruses have been implicated in the pathogenesis of the disease [91–93].

With regards to the relative prevalence of CMV- related AU presentations, we showed in our own series of 38 patients that features of Posner-Schlossman syndrome were observed in 50% of the eyes, Fuchs heterochromic iridocyclitis in 13% of the eyes, chronic nonspecific AU in 21% of the eyes, and corneal endotheliitis in 18% of the eyes [94]. Diagnosis usually relies on aqueous fluid viral PCR or Goldmann-Witmer coefficients positive for CMV [2, 87, 88, 95].

Typically, the differential for AU with ocular hypertension includes herpes simplex virus (HSV), and varicella zoster virus (VZV). There are, however, several signs that can differentiate AU caused by these three herpesviridae. Demographically, patients with CMV-AU are predominately male and older in age. Conversely, mutton-fat KPs are found predominantly in patients with HSV and VZV. With regards to the overall clinical severity of AU and viral load, the highest of both is typically in VZV-AU, followed by HSV-AU and lastly, CMV-AU. Iris atrophy is often observed in HSV-AU and VZV-AU typically with round type and sector type morphology, respectively [96–98].

### Diagnostic implications

Parsing the diagnosis often relies on the distinct differences in anterior chamber reaction. In CMV patients, KPs are common between the entities and difficult to use as a discerning feature. In case of Fuchs heterochromic iridocyclitis, differentials include multiple infectious etiologies among them cytomegalovirus (CMV), and rubella virus (RV). Diagnostic anterior chamber paracentesis typically can be analyzed to differentiate among CMV, HSV, VZV (by Reverse Transcriptase Polymerase Chain Reaction (RT-PCR; Goldmann-Witmer coefficient (GWc)) and rubella (PCR).

### HLA-B27 anterior uveitis mimicking infectious endogenous endophthalmitis

One of the most prevalent etiologies for acute AU is acute unilateral AU related to human leukocyte antigen B27 (HLA-B27) [99, 100] a product of the major histocompatibility complex (MHC). It is a genotype associated with a variety of spondylarthropathies. Classically, patients with HLA-B27 AU present with either an acute unilateral episode, or recurrent, unilateral and alternating episodes [101–103] or less typical presentations of chronic or persistent AU, episcleritis and scleritis [104]. The presence of a fibrinous reaction or hypopion due to severe uveitis in acute HLA-B27 AU obscures the vitreous and retina, and when taken in isolation, can be diagnostically difficult to discern from other uveitis or pseudo-uveitis causes like Behcet’s disease, lymphoma, leukemia or importantly, endogenous endophthalmitis [105, 106]. A Bscan ultrasound can be helpful in these instances and if endogenous endophthalmitis or other infection is suspected, an anterior fluid paracentesis and aqueous fluid analysis should be performed to rule out the differentials.

**Fig. 4.**
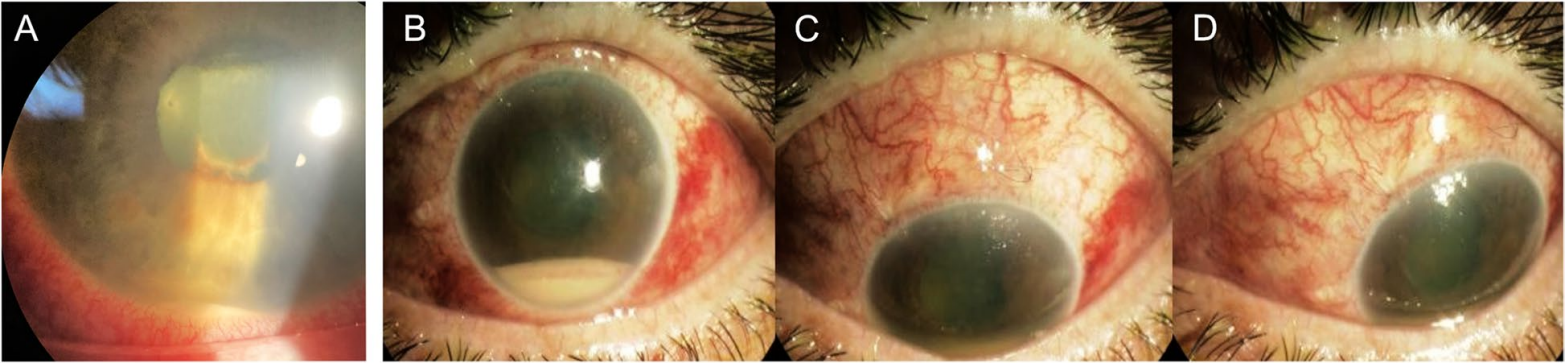
A Slit-lamp photograph of an eye with HLA-B27 associated uveitis. Note the fibrin and sterile hypopyon in anterior segment. **B**-**D** Slit-lamp of an eye post-trabeculectomy associated endophthalmitis. Note the presence of ciliary flush and conjunctival injection

In our experience, and as validated by the literature, HLA-B27 AU with severe anterior chamber inflammation can be misdiagnosed for endogenous endophthalmitis especially in cases where fibrin and/or hypopyon obscure the fundus view (Fig. 4) [107, 108].

Endogenous endophthalmitis describes endophthalmitis secondary to hematogenous spread. It represents roughly 2-20% of all cases of endophthalmitis [109–111]. It classically affects the posterior chamber with findings including retinitis, chorioretinitis, vitreous haze and cell, retinal detachment and retinal periphlebitis. It also involves patients with a history of intravenous drug use that self-inoculate, causing a Gram positive bacteremia. Other etiologies for endogenous endophthalmitis include indwelling catheters, endocarditis or immunosuppression, i.e. HIV/AIDS, diabetes mellitus, chronic corticosteroid use, and malignancy [109–114].

### Diagnostic implications

A patient’s comorbidities can help in differentiating HLA-B27 from endogenous endophthalmitis. Age of onset differs between both uveitidies, with the median age for patients with endogenous endophthalmitis ranging from early fifties to mid-sixties, which is older than for patients with uveitis related to HLAB-27 [109, 111, 113, 115–117]. The symptoms are the same between both uveitis entities. Decreased visual acuity, pain and injection are common, though relatively non-specific symptoms for patients with endophthalmitis [118]. However, definitive diagnosis is typically made with culture from intravitreal specimens [108, 118–120].

### Tattoo associated uveitis

Tattoo-Associated Uveitis is a rare entity that can be often overlooked by ophthalmologists. It is seen in patients with cutaneous tattoos in any area of the body with subsequent bilateral, granulomatous AU. Both the site of the tattoo and the eye are concomitantly inflamed. Currently, the disease is postulated to either be a form of delayed hypersensitivity or systemic sarcoidosis. The overall prevalence is low with its presence documented only in the form of case series [121–128].

Although bilateral, anterior granulomatous uveitis is a more common presentation, it is important to acknowledge that panuveitis with retinochoroiditis, and retinal vasculitis have been documented; along with cases of macular and optic disc edema [126].

### Diagnostic implications

Bilateral anterior granulomatous uveitis immediately prompts a differential of sarcoidosis, tuberculosis, VKH, Lyme and syphilis. In these patients, it is important to elicit a history of recent tattooing and to perform a detailed dermatologic exam.

## Drug related uveitis

Moorthy et al. have provided a comprehensive review of medications associated with uveitis [129] after intraocular injection of antibiotics, urokinase, plasmin/ microplasmin, and antibodies (ranibizumab, bevacizumab, etc.). As such, a non-granulomatous AU was noted in 17% to 89% of cases and hypotony in approximately 10% of cases after the use of cidofovir for the treatment of CMV [129]. There have been several reports of anterior chamber inflammation associated with intraocular injection of anti-VEGF: pegaptanib sodium, ranibizumab (0.3-1.5%) [130–134] and bevacizumab (0.5-1.1%) [132, 135].

### Anti- VEGF medications

Brolucizumab (Beovu) is a novel anti-VEGF molecule, a humanized single- chain variable antibody fragment (scFv). Compared to aflibercept in eyes with diabetic macular edema (DME). KITE, KESTREL and KINGFISHER, phase III studies found an intraocular inflammation in 2.2% to 4.7% of cases with brolucizumab (3 - 6 mg) versus 0.5% to 1.7% with aflibercept (2mg). These studies report a posterior involvement in the form of a retinal vasculitis in up to 1.6% [136–138]. According to the approved label, brolucizumab is contraindicated in eyes with active intraocular inflammation [139]. Phase III trials of brolucizumab for neovascular age macular degeneration (AMD) have also reported higher frequencies of intraocular inflammation [140] (4.6%), including retinal vasculitis (2.1%) and retinal vein occlusion, compared to the aflibercept [140]. Although the cause of ocular inflammation with brolucizumab is unknown, the delayed onset (30-53 days) seems to signal an immune (Type III/IV hypersensitivity), rather than a toxic or infectious cause [140, 141]. Approximately 90% of the uveitis and iritis cases related to brolucizumab were mild to moderate and treated with a course of topical corticosteroids/anti-infectives in phase 3 trials (HAWK and HARRIER) [142]. Subclinical anterior chamber inflammation may occur at rates as high as 20% after intravitreal anti-VEGF injection. Rates of acute onset sterile inflammation have been reported to range from 0.05–2.1%, 0.05–1.1%, and 0.005–1.9% in aflibercept, bevacizumab, and ranibizumab, respectively with clinical manifestations of anterior chamber and/or vitreous cavity inflammation [143]. Many mechanisms have already been put forward to explain the pathogenesis of inflammation following the injection of anti-VEGF medications. These mechanisms can be divided into 3 causes: patient-specific susceptibility, medication specific impurities, non-human proteins, the formulation in which the drug is administered, the immunogenic properties of the actual anti-VEGF antibody itself and protein aggregates may play a role in inflammation caused by these agents [143].

Recently, intravitreal injections of pegcetacoplan (15 mg) to slow progression of atrophy in AMD have shown an incidence of 3% intraocular inflammation, including anterior chamber cells, iritis, and anterior chamber flare, in the DERBY and OAKS phase 3 randomized controlled clinical trials [138].

**Fig. 5:**
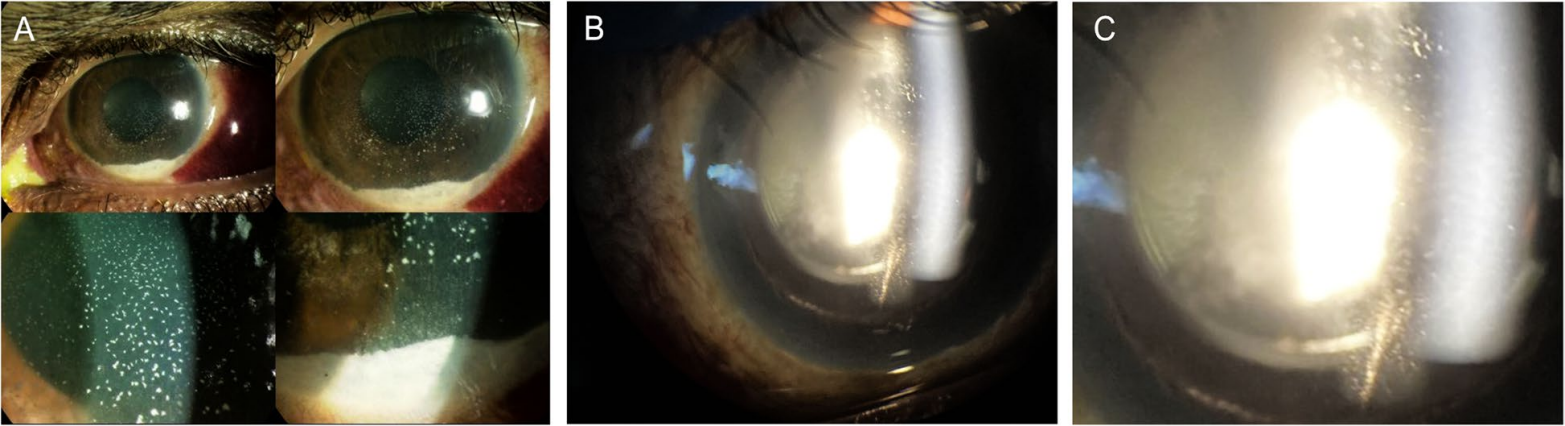
**A** Slit-lamp photograph of an eye with sterile hypopyon following the injection of intravitreal triamcinolone. **B**-**C** Slit-lamp photograph showing the triamcinolone acetonide crystals in the anterior chamber of a pseudophakic eye following intravitreal triamcinolone injection in another patient

It is current knowledge that a severe inflammation following intravitreal injection of triamcinolone acetonide, also called sterile endophthalmitis can happen (Fig. 5). From several reports, Moorthy et al. highlight the substantial anterior chamber cell involvement, often with hypopyon and they evaluated its incidence as occurring in 0.5% to 9.7% of injections [129].

#### Topical glaucoma medications

The same authors have also reviewed uveitis associated with topical instillation of the following: prostaglandin analogs, metipranolol, cholinomimetics, antibiotics, betaxolol, cholinesterase inhibitors. Topical prostaglandin analogs latanoprost, travoprost, and bimatoprost are used to lower the intraocular pressure primarily by increasing uveoscleral outflow have been associated with conjunctival hyperemia, reactivation of herpes simplex keratitis, AU along with cystoid macular edema, iris and periocular skin pigmentation, and iris cyst formation [129]. The mechanism by which prostaglandin analogs might cause AU may involve the downstream stimulation of proinflammatory eicosanoids [144].

Brimonidine tartrate is a selective alpha2-adrenergic receptor agonist that is used for the treatment of ocular hypertension. Of note, there are few case reports describing the occurrence of a granulomatous AU with elevated intraocular pressure associated with chronic use of brimonidine [129].

### Anti-neoplastic medications

Antineoplastics, both specific targeted therapies and immunotherapies can involve all parts of the uvea from anterior to posterior [145]. BRAF/MEK inhibitor therapies are associated with a significant increase in the risk of uveitis, either anterior, intermediate uveitis with or without macular edema, papilledema. The mean probability of developing uveitis during 1 year of treatment has been reported between 0.8% and 5% [145–147]. Similarly, erlotinib, a first-generation small-molecule tyrosine kinase inhibitor which reversibly inhibits the kinase domain of epithelial growth factor receptor (EGFR) and is used in treatment of advanced non-small cell lung cancer has been reported as the cause of AU in a small number of cases [148, 149]. Anterior uveitis alone or with posterior segment involvement including vitritis, cystoid macular edema, sub-retinal fluid, serous retinal detachment or papillitis have been reported secondary to immune checkpoint inhibitor cytotoxic T-Lymphocyte antigen (CTLA-4) and anti PD-1 therapy, each separate or in combination anti-CTLA-4/anti-PD-1 therapy [150–153].

### Others

In their review of medications associated with uveitis, Moorthy et al. implicated the systemic use of sulfonamides, diethylcarbamazine, fluoroquinolones, oral contraceptives, topiramates, rifluoperazine, quinidine, ibuprofen, reserpine, sildenafil, and clomiphene citrate [129]. Among uveitis associated with systemic fluoroquinolone therapy, the most commonly implicated agent is moxifloxacin. The associated AU presents as fine, pigmented KPs with prominent pigment in the anterior chamber with only minimal non-pigmented cell, diffuse iris transillumination defects with atonic pupils and ocular hypertension or glaucoma in up to 50% of patients [153–155]. The pathophysiology of fluoroquinolone-associated uveitis is unknown but Moorthy et al. have hypothesized phototoxicity, autoimmune predisposition, and/or concurrent viral infection [129].

Rifabutin is an oral bactericidal antibiotic used as prophylaxis against *Mycobacterium avium complex*. Rifabutin-associated uveitis is more commonly an AU with hypopyon [129]. The risk factors associated with its occurrence are dosage and duration of rifabutin therapy, low body weight, and use of concomitant medications, including clarithromycin and ritonavir through inhibition of hepatic cytochrome P-450 [156–158]. Moreover, bisphosphonates have been noted to cause conjunctivitis, acute non-granulomatous AU, and scleritis/episcleritis in some patients. Intravenous pamidronate sodium is the bisphosphonate most frequently associated with uveitis [129].

Paradoxical inflammation is more common with etanercept and is much less common with monoclonal antibody type anti-TNF agents [159].

### Post vaccine

Multiple case reports have implicated the following vaccines in the development of acute bilateral AU Bacille Calmette-Guérin (BCG), influenza, Hepatitis B, measles, mumps, and rubella (MMR), diphtheria, tetanus, and pertussis (DPT), varicella, and smallpox [129].

#### Recent post mRNA COVID-19 vaccinations

Numerous reports have been made of cases of anterior, episcleritis, scleritis, but also posterior/ panuveitis after mRNA COVID-19 vaccinations [160]. According to the US Food and Drug Administration Vaccine Adverse Event Reporting System, anterior chamber inflammation (1), conjunctivitis unspecified and viral (6), corneal endotheliitis (1), episcleritis (3), eye inflammation (16), eye edema (1), eye swelling (7), herpes ophthalmic (2), iridocyclitis (33), iritis (13), ocular hyperemia (36), uveitis (155), 851 cases of uveitis after COVID-19 vaccination involved mRNA or adenovirus vector vaccines from the reports processed as of June 24, 2022 [161]. A large multinational case series including 70 patients has shown that the most common events were AU (58.6%), followed by posterior uveitis (12.9%) and scleritis (10.0%). The mean time to event was 5 days and 6 days (range, 1-14 days) after the first and second dose of vaccine, respectively. Among all patients, 36 (54.1%) had a previous history of ocular inflammatory event [162].

In 2022, Haseeb et al. did a retrospective review of 58 studies describing adverse ocular manifestations following any vaccination against COVID-19 between December 2020 and December 2021 for a total of 94 patients included. Of the 87 cases in which vaccine information were present, BNT162b2 mRNA SARS-CoV-2 (BioNTech/Pfizer, Mainz, Germany) was reported 55 (63.2%) times, AZD1222 ChAdO×1 nCoV-19 (AstraZeneca, Cambridge, UK, also marketed as the CoviShield Serum Institute of India vaccine) was reported 20 (22.9%) times, Moderna COVID-19 Vaccine (ModernaTX, Inc., Cambridge, MA, USA) was reported 6 (6.9%) times, BBIBP-CorV (Sinopharm, Beijing, China) was reported 3 (3.4%) times, Corona Vac (Sinovac Biotech Ltd., Beijing, China) was reported 2 (2.3%) times, and Gam-COVID-Vac/Sputnik V (Gamaleya Institute, Moscow, Russia) was reported once (1.1%) [163].

There has been also reports of reactivated uveitis and kerato-uveitis related to underlying infectious disease, herpetic disease, or pre-existing uveitis-related diseases (including ankylosing spondylitis, psoriasis, Crohn’s disease, and herpes zoster (VZV) ophthalmicus) after COVID-19 vaccines [164–171].

Several reports have described complications in the form of conjunctival or ciliary injection, corneal graft edema, Descemet’s folds, cells in anterior segment and scattered KPs involving unilateral and bilateral corneal transplant rejections occurring 2 days to 2 weeks following vaccination to COVID-19 with mRNA vaccine BNT162b2, AZD1222, Moderna anti-SARS-CoV-2 or ChAdOx1 nCoV-19 Corona Virus Vaccine Recombinant, COVISHIELD™ [172–177]. Nearly all types of anterior segment transplantation have been linked with failure in the setting of recent vaccination, including penetrating keratoplasty, descemet membrane endothelial keratoplasty, descemet stripping automated endothelial keratoplasty, and living-related conjunctival-limbal allograft [178, 179].

### Diagnostic implications

The occurrence of AU and less frequently of posterior uveitis or scleritis especially if previous history of ocular inflammatory event (i.e., herpetic, herpes zoster (VZV) ophthalmicus, ankylosing spondylitis, psoriasis, Crohn’s disease) is suspicious of a COVID-19 related uveitis if it happens within a time range of 5 days to 14 days after the first or second dose of vaccine. One might think also of largery prescribed topical antiglaucoma eye drops when AU occurrence. BRAF/MEK/ CHECK inhibitor therapies are associated with a significant increase in the risk of uveitis, either anterior, intermediate uveitis with or without macular edema, papilledema.

Recently, intravitreal injections of brolizumab for exudative AMD and pegcetaplan for atrophic AMD have been involved in uveitis.

## Masquerade presentation of anterior segment inflammation: peripheral ulcerative keratitis misdiagnosed for an infectious bacterial ulcer

Peripheral Ulcerative Keratitis (PUK) is a destructive, corneal inflammatory process. Importantly, it can be either an infectious or sterile process. Infectious etiologies include bacteria such at *Staphylococcus*, and *Streptococcus*, viruses such as HSV and VZV, and fungi. Non-infectious PUK is typically tied to autoimmune systemic pathology such as rheumatoid arthritis, systemic lupus erythematosus, granulomatosis with polyangiitis and polyarteritis nodosa. Corneal ulcers secondary to herpetic infection more commonly mimic autoimmune PUK. Thus, a unilateral PUK in an elderly individual should raise suspicion of herpetic keratitis, prompting the search for other signs indicative of herpetic keratitis, such as endotheliitis or loss of corneal sensitivity. Bacterial keratitis typically occurs in contact lens wearers, with a usually intact cornea and limbus. Fungal keratitis should be suspected in contact lens wearers, patients with chronic ocular surface conditions, or those undergoing prolonged local corticosteroid therapy.

Patients classically present with a unilateral, crescent shaped ulcer in the peripherally cornea, though bilateral presentations are not uncommon. There is often stromal thinning and associated scleritis. Much of the morbidity centers around incomplete treatment which can lead to stromal melting, especially in patients with PUK secondary to systemic autoimmune disease. In patients with PUK and associated scleritis it is also not uncommon to find AU as well [180–182].

### Diagnostic implications

Both PUK and bacterial keratitis can present as peripheral ulcer with patient complaints of redness, pain and photophobia. On exam, corneal ulcers present with conjunctival injection, corneal thinning, Descemet’s folds and stromal edema. Clinical suspicion for PUK is warranted in patients with a history of autoimmune disease, especially rheumatoid arthritis. Patients will often have concurrent systemic symptoms associated with their autoimmune pathology. Contextualizing exam findings with a thorough history inclusive of extraocular symptoms can parse PUK from bacterial keratitis, as well as scraping of corneal ulcer for culture and sensitivity and special stains to rule out infection.

## Conditions that may present as scleritis masqueraders

### Drug induced: biphosphonates

Specific drugs have been shown to induce scleritis. An investigation leveraging data from the National Registry and the World Health Organization found a significant amount of case reports with bisphosphonate induced scleritis [183]. Bisphosphonates have been circumstantially implicated in a diverse range of inflammatory presentations beyond scleritis that are also important to consider [184–188].

### Neoplastic conditions masquerading as scleritis: squamous cell carcinoma, choroidal melanoma, adenocarcinoma

Though largely limited to case reports, neoplastic conditions like choroidal melanoma or undifferentiated carcinoma, conjunctival lymphomas, and ocular surface squamous neoplasia can masquerade as scleritis [186, 189–191]. Corticosteroid-resistant scleritis should prompt a scleral biopsy to investigate for neoplasia. Herpetic origin is also a differential diagnosis for scleritis.

Conjunctival lymphoma has been classically described as a painless, nodular lesion with salmon-pink or fleshy patches at the fornix or bulbar conjunctiva. It is slow-growing, and feeder vessels are not typically seen. Other clinical manifestations such as ptosis, diplopia, or a palpable mass have been reported. Systemic involvement was observed in 20% to 31% of conjunctival cases [192]. The relatively asymptomatic presentation and indolent course of the disease often result in a delay in diagnosis.

Conjunctival epidermoid carcinoma is a rare tumor affecting mainly the perilimbal region of the bulbar conjunctiva that can mimic a Mooren pseudo-ulcer. Conjunctival epidermoid carcinomas typically have a raised and avascular appearance.

### Parasitic ocular infections

Helminth species (e.g., *Brugia* spp., *Thelazia* spp., *Dirofilaria* spp., and *Wuchereria* spp.) may infest human eyelids, conjunctival sacs, lacrimal glands and, in some cases, the ocular globe. Human ocular filariasis, caused by *Onchocerca* species, have been found in fibrous tissue masses underneath conjunctiva mimicking scleritis [193, 194].

### Inflammatory systemic disease: scleritis and intraocular involvement in IgG4-related ophthalmic disease (IgG4-ROD)

IgG4-ROD is a rare progressive disease hallmarked by chronic immune activation across many organ systems including the orbit with resultant tissue fibrosis. The chronic inflammation and fibrosis often cause diffuse enlargement of affected tissues mimicking a neoplastic etiology. IgG4-ROD orbital manifestations include lacrimal gland enlargement, extraocular muscle enlargement and it most commonly presents as inflammation of orbital and scleral tissues, but is also a rare cause of uveitis [195–202].

### Foreign bodies

Non-absorbable sutures from strabismus surgery in childhood have been reported to result in inflamed episcleral granulomas mimicking severe nodular anterior scleritis [203]. Episcleral nodules in the form of lipid granulomas caused by silicone oil leakage near entry sites of vitrectomy have been also reported [204].

### Diagnostic implication

Cases of scleritis non-responsive to treatment should be re-evaluated for a possible neoplastic etiology. A detailed medication review should be performed in initial evaluations of uveitis patients.

In patients with significant conjunctival infiltration, nodular development or chronic relapsing scleritis, IgG4 related disease should be elevated to diagnostic consideration. Diagnosis requires biopsy and histopathologic confirmation.

## Misdiagnosed among scleritis

### Necrotizing scleritis without inflammation (scleromalacia perforans) (differential with scleral hyaline plaque)

Scleromalacia perforans is a rare type III hypersensitivity reaction characterized by the presence of anterior necrotizing scleritis without inflammation. It presents as scleral plaques without significant vascular congestion or injection. It is commonly associated with rheumatoid arthritis [205]. It has also reported in Behcet’s disease [206, 207], ulcerative colitis [208] and graft versus host disease [209]. It can progress rarely to a staphyloma in cases of elevated intraocular pressure. The progressive thinning can lead to eventual spontaneous perforation. Medical management of patients with necrotizing scleritis require systemic therapy with steroids and immunosuppression (often cyclophosphamide or biologic agents), as well as control of associated systemic disease**.** In severe cases with exposure of underlying uvea, tectonic patch grafting may be required [210, 211].

### Diagnostic implications

The difficulty in diagnosis of scleromalacia perforans centers on its indolent appearance. The lack of inflammation allows cases of scleromalacia perforans to be mistaken for senile scleral hyaline plaques. Keys to appropriate diagnosis include consideration of systemic comorbidities such as rheumatoid arthritis. Monitoring of scleral plaques for progression and coalescence can also point towards scleromalacia perforans as opposed to a diffuse scleral hyaline plaque.

### Surgically induced necrotizing scleritis

Surgically induced necrotizing scleritis (SINS) can occur after ocular surgeries with a variable latency period and is diagnosed clinically when ischemia or vascular nonperfusion accompanies scleritis with or without active inflammation. It is thought to be a immune-complex hypersensitivity reaction towards an antigen that is either revealed or altered by ocular surgery [212]. Importantly, cases of SINS have been reported as infectious, predominantly in pterygium surgeries [213]. Although SINS can be a harbinger of underlying systemic disease after cataract/lens procedures, a search for contributing underlying autoimmunity will most often prove unrevealing with SINS following other surgeries, particularly following pterygium surgery [213].

SINS is more commonly associated with the use of antimetabolites, for example, mitomycin C in pterygium surgery or trabeculectomy. However, SINS has been reported after a variety of procedures, including cataract surgery, strabism surgery, corneal grafting, vitrectomy and pterygium excision with conjunctival autograft [207–217].

### Diagnostic implications

Although SINS has been reported after a many surgical procedures, SINS is more commonly associated with the use of metabolites in pterygium surgery or trabeculectomy. Treatment decisions should be informed, therefore, both by the low rate of underlying systemic autoimmunity and the high risk of infection, particularly by *Pseudomonas* and fungal organisms. An infectious origin is the primary etiology to consider in the case of scleritis following ocular surgery. Samples should be taken, and antibiotic therapy initiated.

## Conclusion

In this review, we described the most representative ophthalmologic diseases that manifest with intraocular inflammation that mimic AU/ scleritis, including those belonging to the group of immune-mediated or infectious uveitis entities, classically named as *“Uveitis Masquerade Syndromes”* and we also focused on misdiagnoses among uveitis/scleritis/ anterior segment inflammation entities. In the case of recurrent or persisting ocular inflammation, it is critical to prove the underlying diagnosis if neoplastic (i.e., lymphoma, leukemia or metastatic solid tumors) in order to initiate the appropriate therapy and avoid a systemic spread and consecutive deterioration of the prognosis. Anterior segment infiltration of the eye is an uncommon complication of acute lymphoblastic leukemia and portends a poor prognosis. Systematic reviews underscore the rarity of such cases of anterior segment infiltration.

Early recognition, systemic work-up, and prompt treatment of patients with malignancies with ocular relapse is likely an essential step in improving survival outcomes in these aggressive diseases.

However, other post surgical conditions have clinical features that can also mimic AU or scleritis. If an endogenous endophthalmitis is suspected, the diagnosis must be confirmed by obtaining intraocular (aqueous, vitreous) specimens. Delay of treatment with trial of topical corticosteroids can result in disease progression and worsening of symptoms.

We also showed that previous studies have also reported that HLA B27-associated panuveitis could mimic an endophthalmitis.

The recent reports of cases of AU, after COVID-19 vaccines are particularly worrisome since apart from being potentially sight threatening, this can represent a contraindication on future COVID-19 vaccinations.

A high index of suspicion must be maintained for masqueraders of AU and scleritis, which can include serious inflammatory, infectious, and neoplastic disorders. Scleritis has a broad differential that should be worked through systematically. The differential diagnosis for anterior scleritis includes episcleritis and severe microbial conjunctivitis.

Careful review of possible past uveitis history, current medications and recent vaccinations, detailed examination of signs of past or present inflammation and multimodal retinal imaging are required for the correct diagnosis.

Making the correct diagnosis for AU, scleritis and its masqueraders can be challenging even for the experienced uveitis or cornea specialist, and one must maintain a broad differential initially in order to catch rare entities. The above outline provides insights into distinct clinical features and specific findings on ocular biopsies and anterior chamber paracentesis for cytopathologic confirmation. If anterior segment infiltration is suspected in a patient with an history of malignancy or hemopathy, cytological examination should be performed. Moreover, the use of topical corticosteroids before the first anterior chamber paracentesis may delay cytopathologic diagnosis by reducing the number of tumor cells in the aqueous humor.

## Data Availability

Not applicable.

## Abbreviations

AU: Anterior uveitis
OIS: ocular ischemic syndrome
PIOL: Primary intraocular lymphoma
NHL: Non-Hodgkin Lymphoma
BL: Burkitt Lymphoma
EBV: Epstein-Barr Virus
CVA: Cerebrovascular accident
HIV: Humman Immunodeficiency Virus
TASS: Toxic anterior segment syndrome
KP: Keratic precipitate
TIN: Tubulointerstitial Nephritis
TINU: Tubulointerstitial Nephritis and Uveitis
UWFFA: Ultra-widefield fluorescein angiography
OCT: Optical Coherence Tomography
IRIS: Immune reconstitution Inflammatory syndrome
AIDS: Acquired immunodeficiency syndrome
HAART: Highly active antiretroviral therapy
CMV: Cytomeglaovirus
FHI: Fuchs heterochromic iridocyclitis
PSS: Posner-Schlossman syndrome
HSV: Herpes simplex virus
VZV: Varicella zoster virus
HLA-B27: to human leukocyte antigen B27
MCH: Major histocompatibility complex
VKH: Vogt-Koyanagi-Harada disease
Anti-VEGF: Anti-Vascular Endothelial Growth Factor
EGFR: Epithelial Growth factor receptor
BCG: Bacille Calmette-Guérin
COVID-19: Coronavirus Disease of 2019
PUK: Peripheral Ulcerative Keratitis
SINS: Surgically Induced necrotizing scleritis
